# A qualitative study of the impact of peer support on women’s mental health treatment experiences during the perinatal period

**DOI:** 10.1186/s12884-022-04959-7

**Published:** 2022-09-06

**Authors:** Chloe Rice, Emma Ingram, Heather O’Mahen

**Affiliations:** Mood Disorders Centre, University of Exeter, Washington Singer Building, EX4 4QG Exeter, UK

**Keywords:** Pregnancy, Antenatal, Postpartum, Depression, Mental Health, Treatment, Peer support, Barriers, Access

## Abstract

**Background:**

Perinatal mental health problems are prevalent, affecting up to 20% of women However, only 17–25% receive formal support during the perinatal period. In this qualitative study, we sought to examine women’s experiences with peer support for mental health problems during the perinatal period.

**Methods:**

Semi-structured interviews and focus groups were conducted with twenty-five mothers from the UK who had utilised peer support for a perinatal mental health problem. Data was analysed using thematic analysis.

**Results:**

Seven major themes were identified in women’s help seeking process and experience of peer support. These included; perinatal specific precipitating factors that contributed to their mental health problems, barriers in the form of unhelpful professional responses, feelings of isolation, acceptance of the problem and need to actively re-seek support, finding support either through luck or peer assistance.

**Conclusion:**

Peer support showed promise as an effective means to reduce perinatal mental health difficulties; either as a form of formal support or as an adjunct to formal support. The results highlight ways to improve perinatal women’s access to mental health support through peer-based mechanisms.

**Supplementary Information:**

The online version contains supplementary material available at 10.1186/s12884-022-04959-7.

## Background

Perinatal mental health disorders are highly prevalent and disabling, affecting up to 20% of women [1] with long-term potential negative impacts on the mother-infant relationship and child social and emotional outcomes [2]. Although there are efficacious perinatally-adapted treatments for mental health disorders that occur during pregnancy and the postnatal period [3], only 17–25% of perinatal women will receive formal support [4] compared with 40% of those with a mental health problem outside the perinatal period [5].

A substantial body of research has now demonstrated that pregnant and postnatal women face a number of critical barriers to accessing appropriate mental health support, even when it is available [6–8]. These barriers may be either woman centered and include both practical and psychological barriers; e.g., childcare difficulties; not understanding the problem, and stigma [8, 9] or originate within the healthcare system, and include lack of systematic screening for mental health problems, and poor referral rates. Thus, even if women attempt to overcome their personal barriers and ask for help, they are likely to encounter barriers to accessing support within the healthcare system itself. Despite efforts to reduce barriers to mental healthcare receipt, such as ameliorating practical barriers (e.g., childcare provision, transportation vouchers, remote treatment) or healthcare barriers (e.g., universal depression screening) there has been little sustainable and systematic improvements in women’s perceived access to care [10]. Innovative strategies that are scalable, have low-stigma and are acceptable are therefore required.

There has been increasing interest in the role of peer support in both augmenting mental health treatment and in supporting women’s access to and engagement with perinatal mental health treatment. Peer support is defined as the exchanging of resources and shared discussion of experience between like-minded, relatable peers with similar experiences [11]. Amongst a number of possible roles, peer supporters may help to improve the scalable delivery of interventions, be an acceptable and cost-effective adjunctive source of support in complex interventions, and increase service outreach to populations with poor treatment engagement. Outside the perinatal period, recent narrative reviews of peer support interventions have found that they may offer some benefit in terms of improving retention with existing mental health programs and peer staff delivering structured curricula [12]. Existing research on peer support in the perinatal period suggests that women find peer-support acceptable and that it provides them with important mental health benefits [13, 14]. For example, women reported feeling less isolated, more confident as mothers, and more likely to seek or continue with formal mental health support [13, 14]. In recognition of the potential of these roles and benefits, there has been growing international interest and investment in the peer support role across a range of service contexts [12, 15]. Despite this, however, the evidence-base on where peer supporters may most effectively fit within perinatal mental health service delivery remains unclear. Most of the research on peer support during the perinatal period has focussed on evaluating specific, formal interventions provided directly by peers, and the efficacy of these interventions has been mixed [16–20]. In contrast, there has been little research examining the other adjunctive or informal functions of peer support which are often widely available through charities, non-profit and non-governmental organisations, despite the fact that a recent review outside the perinatal period has found that these latter functions may offer greater clinical benefit [12]. These functions may focus on providing social support, reducing isolation, and helping women to effectively navigate healthcare systems. Understanding the range of possible processes through which informal peer support functions may help to identify scalable, systematic ways to improve perinatal mothers’ engagement and adherence with formal treatment whilst also sustaining recovery post-treatment.

We therefore aimed to investigate women’s experiences of mental health support during the perinatal period and the role that peer support had in those experiences.

Additionally, we sought to examine and contrast women’s barriers to accessing peer support versus formal support and investigated the adjunctive roles informal peer support served alongside formal perinatal mental health. Using qualitative methods, we asked, “What are women’s experiences of peer support in receiving mental health peer support during the perinatal period?”

## Method

### Participants

Participants were 25 mothers residing in the South West of England and South Wales.

Individuals were eligible for the study if they were 18 years of age or older, currently engaged with peer support or had been within the last 3 years, had experienced a mental health problem that caused them significant distress and/or negatively affected their functioning while they were either pregnant or in the first postnatal year, and were able to speak and understand English. Women who were actively psychotic or substance dependent were excluded from participation.

Participants were recruited either via third-sector organisations or Facebook groups. Non-profit organisations working with women matching our inclusion criteria emailed women details of the study and informed consent documents, or provided them with links to a Facebook group that advertised study details and arranged focus groups or invited individuals to participate. Snowballing strategies, with participants referring other participants, were also used.

### Procedure

Ethical approval was received by the University of Exeter (eCLESPsy000150). Written informed consent was obtained from all participants. All methods were performed in accordance with the Declaration of Helsinki guidelines and regulations. A qualitative interview guide was developed by the research team following a review of the literature, researcher discussion (HOM, clinical psychologist), and input from stakeholders (a non-profit provider and a woman who had used perinatal mental health and peer support services) (see Online supplemental materials). Semi-structured Interviews were conducted in either four face-to-face focus groups (*n* = 18), or, for those individuals unable or unwilling to attend the focus groups, six individual interviews were conducted. Focus group methods were used to highlight a broader range of topics, whereas later individual interviews were used to help gain greater depth of content on the interview topics from participants. Interviews lasted between 40 min and 1 h. The interviews focused on women’s experiences of seeking and receiving mental health treatment, specifically within peer support provisions. Women were asked about how they became engaged with services and specifically, peer support, the suitability of the content and delivery (e.g. location, childcare etc.) of the interventions they received, the quality of different forms of support, and what factors they perceived to be beneficial to both their acute and ongoing well-being. All interviews were conducted by either the first or second author, under the final author’s supervision. Interviews were audio recorded and transcribed verbatim.

### Analysis

Data was analysed using an iterative process of inductive and deductive thematic analysis, as outlined by Braun and Clarke [21]. The first two authors read the transcripts several times to become immersed in the data. Initial codes were independently generated line-by-line. These were then iteratively discussed with the final author, until consensus on the coding system was derived. The first two authors then undertook cross-coding of a subsample of interviews to ensure coding reliability. An initial thematic model of themes and subthemes was then created in discussion with the final author. The interview guide was iteratively amended as new questions emerged from the themes. The final themes were derived following re-reading and cross-checking codes and after team consensus. The language for code labels was derived from participant responses (e.g. “Secret Society”), the broader perinatal literature, and professional clinical understanding (e.g. “Effective Triage”). Focus group and individual responses were compared, and where individual responses provided greater contextual detail, or disconfirming data, this was highlighted and weighted alongside the focus group data. This process of rigorous coding, in-depth discussion and reviewing each transcript multiple times enhanced the credibility of the model.

### Reflexivity

The researchers were mindful throughout the coding and analysis process of their positioning, background and experiences (i.e., working in services, trainee clinicians/perinatal clinician, personal knowledge of close others who sought mental health support) and how these experiences might have influenced their interactions with participants and analysis of the data.

## Results

**Fig. 1:**
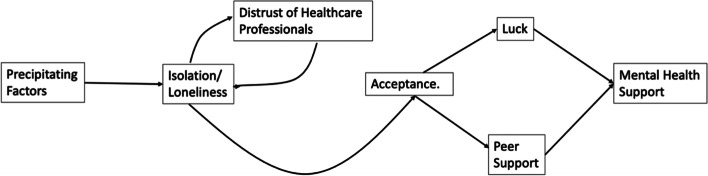
Model of women’s mental health treatment journeys and when and how peer support influenced treatment access

Women described superordinate help-seeking themes that together formed a process of help-seeking which included peer support ((‘Precipitating factors’, ‘Distrust of professionals’, ‘Assumptions and being unheard’, ‘Acceptance and Luck’). This process was characterized by factors that both facilitated and hindered effective engagement with support (see Fig. 1).

Overall, women described the help-seeking process as difficult; “It does feel like… mental health support is like a secret society,” (M7). Women reported that the help-seeking process started with individual factors that affected their motivation to disclose their problems, but quickly this process interacted with external factors to determine whether and how they were able to access and benefit from care. Many women noted that peer support made a key difference in their ability to navigate and engage with perinatal mental health services.

### Precipitating factors

Women reported that their mental health journey started with factors that both precipitated their mental health problems and contributed to their difficulties seeking mental health support. These precipitating factors contributed to feelings of isolation and loneliness that the majority of women reported were key factors underpinning their mental health problems. Their loneliness also prevented them from seeking out mental health support from what they perceived to be a complex and confusing care system. Women described how their loneliness was brought on by judgement from others, worry about their baby, their own physical health issues, disillusionment resulting from unmet expectations, and practical difficulties getting out and about with a new-born baby. They reported feeling that their circumstances were different from other women’s experiences and described feelings of shame about their personal responses that further compounded their sense of loneliness. This sense of shame about being “different” then contributed to feelings of self-blame and prevented women from admitting their difficulties to others, including health care professionals.

#### Worry

Worry was a prominent theme across the perinatal period. Women reported worries that something might go wrong with the pregnancy or their infant’s health, that their mothering was ineffective and could cause long-term problems for their infant, and rumination about difficult childbirths.

*“The pregnancy and the birth um you know it was really tough and there was probably about a year there that was really tough and most of its because it was isolating.” M6*.

*“The initial pregnancy was absolutely awful I felt like I was having a breakdown with fear, I thought I was having a miscarriage just bleeding all the time… ummmm so yeah my mood was initially terror and then it was just kind of getting on with everything.”M2*.

*“I just went into total hibernation, even going to the supermarket was too much. I just didn’t want to see anybody.” FG2/M2*.

Women described how these worries interfered with their abilities to connect with their baby and other mothers and contributed to their sense of loneliness.

*“I wouldn’t leave my daughter even to go to the toilet because I thought she was going to die if I left the room and I couldn’t see that I would ever ever not feel like that” M3*.

#### Physical health problems

Some women reported that physical health problems, during pregnancy, as a result of difficult birthing, or related to breastfeeding, contributed to feeling alone and different, in addition to being a practical barrier to seeking mental health support.

*“Yeah so I had hyperemesis gravidarum while I was pregnant… I was so depressed, I was so ill, I didn’t feel like I could carry on with my pregnancy.”M3*.

*“I bled from 6 weeks and that carried on until week 16 so that was very frightening and then had a breast tumour at 17 weeks pregnant and ended up having two lots of surgery for that… everything spiralled out of control with the breast tumour and fear” M2*.

*“I was desperate, desperate to breastfeed him and um when I got home I just couldn’t do it, I just couldn’t do it. I got to the stage where I was just like I am not coping, I am not dealing with this.” FG3/M1.*

Mothers also reported that because many healthcare professionals were baby-focussed, they were unable to adequately discuss their physical health concerns, and subsequently felt that their mental health difficulties merited even less attention.

*“I remember the midwife saying, you must be bonding with your baby ok and you look like you’re doing alright and I just went along with it” FG4/M2.*

*“You basically go into hospital given a baby and then they check you over they say right see you later and then you’re flushed out into the community and then … that’s where all the problems start.”*

#### Unmet expectations

Women often reported their internalised societal views of motherhood were not met (e.g., not feeling bonded with baby). Unable to describe this to other mothers, or feeling that their experiences were different from others’, they reported feeling isolated.

*“…like that initial bit when I’d pictured me holding him and stuff didn’t quite happen and then I just. I didn’t … I expected… I know it’s like you shouldn’t expect anything, but I expected to feel like oh my god I’m a mum, really excited and stuff… and I didn’t I just felt really, really, rubbish.” M1*.

*“Everyone the whole time is like, ‘oh it’s so exciting’ and then especially, when you’ve just given birth, and I had such a difficult time, that every single person just says to you, well at least you’re okay and at least you’ve got your baby” M3*.

### Distrust of professionals

Faced with feelings of loneliness and isolation, and a growing sense of shame, women reported a reluctance to disclose their problems to health care professionals, and this was compounded by not knowing who to disclose to, nor what help might be available.

In this vulnerable position, requests for help were typically only made once women felt they were past a point of being able to manage without support. Despite the effort it took them to ask for help, many women reported negative or belittling responses from healthcare professionals that further increased their sense of shame and isolation.

#### Knowledge barriers

Faced with worsening feelings of isolation, loneliness and mental health problems, many women described attempting to disclose their difficulties to health professionals, sometimes at the prompting of family members. Most women reported that these initial attempts were difficult to undertake. They described not knowing how or where to seek support, and the consequences of seeking support were also unclear; women expressed fears that their baby would be taken away.

*“If you don’t have that information you feel like there’s no-one you can turn to and it’s seeking it out for yourself which in that moment can be really hard” M4*.

*“Some sort of reassurance that if you admit there’s a problem, your baby’s not gonna be taken away or you’re not gonna be put on some sort of list” FG1/P5*.

Aware of the stigma surrounding mental health, they worried about accepting a mental health problem as part of their identity, and were fearful that seeking support would confirm they were not managing motherhood. Despite this, the ongoing distress they were experiencing prompted them to disclose, though most reported these initial efforts were typically unsuccessful.

*“I just, I didn’t know what was on offer, I didn’t really… I was just being passed around so many people and having to explain everything to strangers so many times… it’s really hard and really exhausting.” FG1/M3*.

#### Assumptions and being unheard

Women reported that they found it difficult to overcome health-care professionals’ assumptions that a mother’s mood is naturally good, and that their worries were related to ‘new-mum’ anxiety and would eventually dissipate. If healthcare professionals conducted screening for mood problems, women described these exercises often felt like healthcare professionals were “ticking boxes” rather than demonstrating genuine care.

*“A doctor said to me do you not think the issue is you, do you not think you’re being over the top because you’re a first time mum?” M6*.

*“The first person said that I seemed bright and bubbly and I’d managed to put my makeup on, that was his actual words, so I must be fine.” M3*.

*“You know and it’s hard but a professional doesn’t necessarily ask you or if they ask you they say it very blasé like oh we assume your mood’s okay yeah? And then they move on to the next question” M6*.

This was evidenced, to women, by healthcare professionals failing to further examine problems women might mention during the screening.

*I just felt a very quick rushed appointment and to actually say no when they were just expecting to be able to tick a box - I just went along with it because it just felt like they wanted me in and out.”FG4/M2*.

*“Until I actually gave birth I really hated her because no one would let me talk about how I felt about being pregnant. And she was like ‘why haven’t you told me’ ‘I’m like you didn’t me a chance so for her just to see how bad I was and just breaking down in front of her”” FG1/M4*.

#### System strain & failures

If healthcare professionals did take account of women’s mood, women reported that downstream plans for managing mood problems were frequently lost because of poor documentation of the problem and a lack of collaborative working between healthcare professionals. Women described having to repeatedly re-explain themselves to multiple professionals.

*“when I spoke to them they said did you know you could have self-referred ages ago. rather than wait for the doctor because I felt like I did say to the doctors a couple times about it and health visitors.” M6*.

Sometimes women discovered too late that healthcare plans had not been accounted for (e.g., in the middle of childbirth, plans for support were undocumented and unavailable). These system failures provoked feelings of betrayal and contributed to negative feelings towards the system, predominantly mistrust.

*“Obviously I had the (childbirth) plan, and then I was with a … midwife, but then none of that actually came to be.” M3*.

*“Not a single person who I was promised… would support me through it, not a single person came to my birth” M3*.

A few women reported that with repeated efforts they managed to get referred to an appropriate mental healthcare service, only to then face long waitlists. They reported that their interactions with formal health and mental healthcare systems compounded their anxiety and sense of loneliness and isolation, which led to deepening feelings of hopelessness.

*“I get really angry about it, just about how I feel like the system has failed the mums who struggle afterwards” M3*.

### Acceptance

Following their frequently unsuccessful initial attempts to seek out formal support, women reported a period in which they retreated away from formal healthcare professionals.

*“You can deny it if you just don’t say it.” M6*.

As their emotional health continued to deteriorate, however, they reported they began to accept that the difficulties they were facing were unlikely to resolve on their own, and required more intensive support to recover, regardless of the responses they’d initially received from health professionals.

*“Because I know for a fact that I didn’t like to think aw I’m one of the statistics that ended up with depression and post-natal depression so sometimes it can be quite intimidating to admit something like that.” M5*

*“A lot of the problem was accepting this whole other life” FG4/M3*.

By this point, many women realised they could no longer seek out support by themselves.

*“I um I sort of broke down at one point and said to my partner how can I tell people that I feel this bad when I can’t actually say it myself”M6*.

They described feeling too hopeless and exhausted to adequately describe their emotions and to actively “fight” to get needed support. Women frequently noted that at this point they needed someone to ‘take charge’, finding self-referrals difficult.

*“I thought, I don’t know I’ve got the words to self-refer can you please do that for me, and that felt massive asking her to refer me for something.” FG2/M2*.

### Luck

#### Secret society

 Some women reported that if they were eventually successful at getting formal mental health support on their own, it was typically down to luck, rather than being able to reliably and systematically being able to access help. Many women stated that to get to formal mental health support they needed to be “invited”, labelling it a “secret society”; available only to the privileged few.

*“It does feel like when you’ve given birth that mental health support is like a secret society.” FG4/M2*.

*“It’s almost like you have to be invited.” FG3/M1*.

*“I only know about the children’s centres because my mum works there.” M5*.

*“The only thing that is wrong is that people don’t know they’re there.” M6*.

To access this secret society, they felt one needed the luck of having a relationship with an empathic healthcare professional who seemed to genuinely care about them, and who had the knowledge and persistence needed to help them advocate for access to the ‘secret society’ of mental health support. Finding such a person often required asking for help from a range of health care professionals.

*“And then she read my medical files and saw that I was diagnosed with mental health problems and I’d had quite an extensive history and quite extensive treatment, and she referred me to the perinatal team.” FG1/M1*.

Once women accessed support, they reported it was easier to access further support.

#### Location and timing

Women reported that their access to mental health support was also affected by the area they lived in and what was offered in that area, and whether or not they had the “good luck” of delivering their baby at a time that corresponded with the start of support groups.

*“As having a July baby…there was nothing -- It’s a big black hole… I was just sort of getting confident to leave the house and suddenly like there was nothing on, and you’re just like ‘oh ok, now what you gonna do.” FG1/M5*.

*“I [couldn’t] access… the buddy system because I’m too far out.” M1*.

### Effective peer support

In this context of isolation, confusion, and frustration in trying to seek help, peer support helped to equalise imbalances in accessing formal mental health support due to luck. Women noted peer support was easier to access than formal mental health support because it was more visible, had fewer barriers to access (i.e., fewer exclusion criteria) and felt less stigmatising. The nature of peer support was also more varied and flexible than formal support. For example, women reported peer supporters might provide outreach and daily reminders to engage in activities, host group-based baby-friendly activities for mothers (e.g., mental health skills courses alongside knitting/art/informal coffee chat groups) and would provide advice and input about how to navigate formal mental health support, including buddying services. Typically, these provisions were group sessions with around 10 to 20 women attending. They felt that the range of these peer support activities reduced their sense of isolation, helped them to rebuild a needed daily routine, and supported them in accessing and adhering to professional support. For some women, the practical and inclusive nature of peer supported activity groups and one-to-one support was enough to help them find a path to wellness. For others, these activities and support provided a foundation that helped them to find the internal strength to try to re-navigate the formal mental health network. To that end, having peer supporters alongside who had knowledge of the system and what it felt to be a new mother struggling with mental health problems while trying to navigate that system, proved for many to be a critical combination that gave them the hope they needed to access professional help.

#### Purpose and focus

Women noted that peer support was most effective when it had a purpose or focus. They reported that peer support worked better when it was regular, practical in nature and worked towards mutually agreed goals (see Table 1 for service recommendations). Women described that this focus worked particularly well in two domains: outreach support and group-based activities. For example, they described reminders from peer supporters to engage in planned activities helped them to stick with plans and routines. Women reported that peer led group activities gave them structure in their day and helped them to develop new skills that they felt positive and efficacious about.

**Table 1:** Recommendations for future initiatives based on women’s desires – with quotes to demonstrate

Bridge Gap/Peer Navigator	Peer Support Direct Mental Health Support
**Peer Navigators to help mothers understand potential different mental health offers.** *E.g. FG1/M5: “I don’t think what anyone should do is say ‘we’ll ditch these types of expensive support and use social media’, That wouldn’t replace face-to-face whatsoever, but I think that this is a channel that’s not being used as much as it could in support.” * **Funding for children’s centers/local community parent-infant groups with regular peer support provision and occasional health visitor input.** *E.g. M6: “I used to go to the children’s centre but they don’t have that anymore, you don’t just drop in anymore – and obviously with cuts it’s gonna keep changing.” * **Joint drop-in sessions with peer supporter and midwife or health visitor.** *E.g. FG2/M5: “There might be something you think I just need advice on, or something like a rash you might be worried about taking them to the doctors, or you’re wondering about how much they’re feeding, obviously we can only give advice on what we know ourselves,* *having a professional opinion would be quite nice.” * **Ensuring healthcare professionals who are in contact with mothers are aware of local charities and resources – Effective and earlier signposting.** *E.g. M5: “No one really knows about it either so if there was someone needing help and like for me the professionals didn’t pick it up, they might not necessarily get the opportunity to go.”* *FG4/M2: “You know just get this information so you’ve got it and you don’t, you might not necessarily need to use it after you’ve had your baby but at least you’ve got it in your house.”* This can be broken down to resources regularly updated and given to mothers at two different time points: booking the birth and delivery. Similarly, advertising services in multiple locations such as GP bulletin boards and websites.	**Groups more accessible to working mothers / mothers of toddlers and older children.** *E.g. M5: “So I think it would be better for it to be open for maybe toddlers as well like if people got toddlers because I don’t believe that post-natal always happens straight away.” * **Out of term-time groups.** *E.g. FG3/M5: “I forgot that. As having a July baby.. there was nothing – it’s a big black hole.. I was just sort of getting confident to leave the house and suddenly there was nothing on, and you’re like ‘oh ok, now what you gonna do’.” * **Specialised & regular monitoring of official peer support groups** *E.g. M3: “So I mean the Facebook group is helpful, it’s quite difficult because obviously there’s so many people in it and it can actually cause some problems, because you know as any kind of chat room or anything people can have like little arguments and stuff inside of it, people can say stuff that’s insensitive.”* **Expanding support/offer of Third- Sector Organizations working in perinatal mental health** *E.g.: FG1/M1: “I don’t think (formal healthcare service) can - I don’t think they can cope with the amount of people who need the service right now.” * *FG1/M5: I think it’s unanimously: more (third sector service)! More funding for (third sector service)!” * **Ensuring facilitators are trained to cope with managing endings of groups and keeping a focus.** *E.g. M1: “I found it a really depressing and unhelpful group – the woman would only pick quite a loose focus and they were never focused.. quite often she would pick something that you could speak about negatively and all it really turned into was a group of women sat round talking about how rubbish stuff was.” *

*“Every week you were working on something and every week are you going to come out thinking I’ll carry that on” M1*.

*“I think most people who come here, just coming here is what they need, just to get out of the house, talk to other people about the same issues, just have a break for a little while and have a hot cup of coffee, I think … it just boils down to something that simple.” FG2/M5*.

Women said that these activities helped them to focus on matters outside their head, which reduced rumination and increased their motivation and sense of achievement. Morning groups were regarded as especially helpful as women started their day productively, rather than becoming caught in a cycle of avoidance.

*“I think it was always better that it was in the morning because it gets you up and it gets you ready and it gets you out” FG4/M1*.

#### In it together

The shared lived experience of perinatal mental health problems of both peers and group facilitators appeared to be a vital aspect of peer support. The knowledge that others had faced similar challenges and could relate to their feelings reduced women’s fear of judgment and validated them in times of difficulty or high emotion. Women also met mothers at different points of their journey; seeing that others had overcome their struggles gave them hope.

*“Um so the best thing about the peer support is that other people, real people, have been through the same thing, so you don’t feel so alone, and there are people on different stages of your journey”. M3*.

This ‘In It Together’ aspect also encouraged reciprocal helping, as mothers felt genuine concern towards other mothers, something that they felt lacked in professional support.

*“The other groups I went to I didn’t really feel like… they were quite clear(ly) not understanding of the mental health issues I was going through. But here you could be around people that understand it” FG1/M4*.

*“… safe environment you can express all those feelings and that really helped me the most was being able to say all that stuff you’re just too scared to say to anybody else” M3*.

*“They can say no I was exactly the same I used to do this, and that kind of validation makes you so much better, that actually would reduce my anxiety loads.” M3*.

Not feeling different led to more positive interactions with themselves, others, and their baby. It also helped them to gain perspective on their problems, realising that they might have more control over their situations than they had imagined. This was true for both face-to-face and online support.

#### Connection correcting loneliness

Alongside the practical, activating and validating nature of peer support, women reported that they made deep and genuine friendships with other similar mothers in the activity-based groups peer supporters led. These friendships were sustained outside of the group and had a profound impact on women’s loneliness. They developed strong bonds of trust that reduced their anxieties as they had a dependable source to share problems with. This created consistency in an inconsistent time.

*“I think most people who come here, just coming here is what they need, just to get out of the house, talk to other people about the same issues, just have a break for a little while and have a hot cup of coffee, I think that is, it just boils down to something that simple.” FG2/M5*:

*“It was good to connect to other people and I can text these guys at any time saying I’m struggling and they understand” FG3/M2*.

*“I think cause they wanted to help me, I wanted to help them” FG3/M5*.

*“when you’re not feeling okay to get hold of [professional support] we know we have [group name] …,groups like that you would be able to make an appointment” M6*.

Connection with babies was also positively-reinforced. Many women described group interactions as increasing their confidence in their parenting abilities (see Table 1 for recommendations).

*“You could be around people that understand it and they just like helped me bond with her and play with her and learn how to enjoy her.” FG1/M4*.

*“… a nice thing to interact with your baby because you don’t necessarily do it at home.” FG4/M3*.

#### Navigating the healthcare system

Peer supporters provided mothers with critical knowledge and support about how to navigate the healthcare system and supported them in advocating for their needs (see Table 1). Peers provided mothers with insights on helpful treatment options and ways to access these treatments, increasing mothers’ sense of efficacy. At times, peers helped to introduce them to group sessions when they were anxious. Further, mothers said that peer supporters gave them the knowledge that with persistence, appropriate support could be available.

*“When you’re not feeling okay to get hold of [professional support] we know we have [group name] … groups like that you would be able to make an appointment” M6*.

With encouragement from peer supporters, and with a newfound sense of having a strong base of peer support, women who required formal mental health treatment reported they were able to sustain their attempts to engage with and adhere to treatment. Suggestions for peer support, based on women’s interviews, are included in Table 1.

## Discussion

Peer support in the perinatal period was described as an effective, positive, dependable support for women, helping them to overcome health care system barriers that contributed to their mental health problems and improved their ability to access mental health support. Crucially, the flexibility of peer support and the positive, de-stigmatising focus it provided was highly valued in comparison to the formal support sector, which women described as rigid and structured. The results suggest that peer support may serve a role in providing perinatal women with consistent, acceptable, accessible, scalable adjunctive support and be a key mechanism through which to overcome common barriers to formal mental healthcare support. These results are consistent with a growing literature demonstrating that peer support is an important growing workforce that may have particular strengths in increasing the reach and quality of mental health support [22].

In this study, perinatal women, already struggling with feelings of isolation and loneliness, reported that finding and engaging in mental health support was like joining a “secret club” marked by their poor knowledge of what might be available, how to access it, and health professional “gate-keeping” to mental health services. These findings are broadly consistent with reviews of qualitative studies examining perinatal women’s reports of barrier to accessing mental health support [6]. In this study, women stated that the journey to joining this secret club was arduous, requiring levels of persistence, assertiveness and sustained motivation that many women felt they lacked. Consistent with previous research [6, 9], women found healthcare professionals were frequently invalidating, and failed to follow-up on women’s mental health needs with referrals or treatment plans. In contrast to these negative experiences, peer supporters helped women to effectively navigate formal mental health services while also providing them with direct mental health support. These descriptions build on research that has demonstrated mental health peer-navigator roles can increase the reach of mental health treatments to individuals with serious mental illness [23] and improve their engagement with and adherence to formal mental health support,^31^ suggesting this role may help bridge important gaps in perinatal mental health service provision, alongside continuing mental health awareness education and collaborative health and mental health care. Further, they support nascent research indicating peers may have a direct mental health support role [18].

Critically, women described key mechanisms of both peer navigation and direct peer support that supported both their own mental health, their parenting confidence, and their perceived relationship with their baby. Incorporating these mechanisms into existing health-care systems may further improve treatment access and engagement with services [24]. Across all forms of peer support, women reported core mechanisms that included connection between women, normalisation of feelings, and validation [25]. Women also valued having child-friendly destinations to go where they could engage in purposeful and structured activities with other mothers struggling with similar mental health problems. They reported these activities helped them to combat isolation and rumination. Women described unstructured activities and support as less helpful. These mechanisms align with current literature on the importance of shared group identities in providing individuals with purpose and motivation and ameliorating loneliness associated with the loss of shared identities during periods of transition (e.g., moving from “employee” and “friend” identity to “parent” identity) [26, 27]. They are also consistent with the behavioural literature, which highlights the importance of routine, structure and focus in guiding individuals from avoidance and low mood states towards engagement with meaningful goals [28] and build on research in substance abuse and mental health services that note that peer support can increase service user activation [24]. Notably, women stated that they preferred peer support that was specific to mental health over generic forms of parental support, as they felt the former was ‘safe’ and helped them to face and overcome stigma-based concerns. This suggests a critical role for targeted forms of mental health peer support during the perinatal period.

Lastly, women reported they appreciated having flexible ways to access mental health support, from online peer support and moderated internet chat-rooms, to drop-in groups or individual sessions about targeted topics, to regular, structured contact with peer supporters or engagement in activity-based groups. They noted the importance of having a range of supports available, being able to bring their babies and older children along, and critically, having choice about what they wanted to access and when. This approach matches with recent patient-led approaches used widely in Finland [29], and suggests that integrating mental health support alongside existing parental offers (i.e., parent-child activities) that mental health peer-supporters might also facilitate, may extend the range of accessible offers to new parents. Suggestions for specific forms of clinical support, based on women’s recommendations, are included in Table 1. Regarding cost, women noted that a number of these services required relatively low input from the supporter (e.g., chat-room monitoring) and often could be used in a less rigid and less intensive way (e.g., 1x/month).

Peer support was not always perceived to be effective, and in some circumstances, where peer supporters did not have appropriate training or supervision, mothers commented its less useful nature. These findings are consistent with the mixed nature of research on peer support in the perinatal period. When well-trained, resourced and supervised, peer supporters can be an accessible and effective workforce [18], but fail to effectively support mothers without these forms of oversight in place [16, 17, 19, 20].

### Strengths and Limitations

We investigated a range of peer support types of provision across multiple non-profit charities. This allowed us to compare which components of peer support were perceived as most helpful. Although the study was conducted across a large geographic area, it is an area marked by low ethnic diversity. All participants were of White British ethnicity, limiting generalisations from this study to more diverse populations. However, it is notable that even in this population, women described mental health support as hard to access. Given even lower treatment access rates amongst minoritized groups [30], it is critical to more widely examine whether peers may serve especially important roles bridging the gaps between minoritized women and mental health support. This study also suggested that peer navigator roles may be particularly useful in helping women to not only engage, but also adhere, to mental health support. Future research examining when, for whom and how such roles are effective is needed.

We did not gather diagnostic information from women, although all of the women in the study experienced mood, thought and behavioural problems that were distressing or impairing enough that they persisted in seeking support, with all of them engaging in peer services and some with formal mental health support. However, it is not possible to say whether women who suffered from specific, diagnosed mental health problems may have benefitted differentially from peer support. Also, the women we interviewed were all engaged with peer support services. We therefore were not able to reflect the views of women who may have not found peer support services acceptable or useful. All of the individuals we interviewed were women. Although there are a growing number of services available to both mothers and fathers, or specifically to fathers, we did not have any fathers respond to our invitations to interview. Lastly, all the mothers in this study were post-partum biological birth mothers of their children. Additional research examining the views of pregnant, non-biological, same-gender mothers or birthing persons who do not identify as women is needed.

In conclusion, trained and supervised peer support shows promise as an effective means to reduce perinatal mental health difficulties; either in itself or as a peer-navigator with formal support. Peer support may be a critical mechanism through which to ameliorate the inconsistency in professional responses to perinatal mental health. The focussed approach peer supporters have on mental health and parenting may overcome barriers healthcare professionals have balancing joint attention on parent, child, health and mental health.

## Supplementary Information

**Additional file 1.**

## Data Availability

The datasets generated and analysed during the current study are not publicly available because it is qualitative data that is personal in nature in its entire format. To protect the confidentiality of the participants, the data are available from the corresponding author on reasonable request.
