# Supplementary material for: A qualitative study of the impact of peer support on women’s mental health treatment experiences during the perinatal period
Source: BMC Pregnancy Childbirth. 2022 Sep 6;22:689. doi: 10.1186/s12884-022-04959-7 (PMC9450402; doi:10.1186/s12884-022-04959-7)
Supplement: Supplementary file 1 — Additional file 1. [file 12884_2022_4959_MOESM1_ESM.docx]

**Supplemental Figure 1:**

*Interview Guide Flow Chart for qualitative interviews*

**Summary**

What would you like to see in terms of third-sector organisations peer support groups in terms of delivery?

Links to NHS support – what would they want from NHS/peer support? Can peer-support support NHS care?

Would you like to see more flexible appointments/locations?

Would perinatal self-help resources be beneficial to the groups?

The overall usefulness of peer-support groups

What would you like to see peer support groups offer to perinatal women?

Any positives or any areas for development?

**Access**

The moment you knew you needed support

How were you referred to the peer-support group? – Wide number of recommendations?

Welcoming nature of the group? Initial feelings towards going to support group?

How quick/efficient was this process?

**The peer support group**

How suitable was the content of the support for your problems?

Quality of perinatal knowledge from support-giver?

Useful resources? e.g. self-help guides

What was the quality of experience?

Most helpful parts?

**The Delivery**

How suitable was the support group? e.g. face-to-face, over telephone, social media support?

How suitable was the arrangement procedure - e.g. timing of groups, booking?

Was there childcare support at group?

Was the group flexible? e.g. accessibility/baby-friendly

**1^st^ section of interview**

Tell me about your experience of; pregnancy

-(any mood changes throughout)

-the period after giving birth
